# *Mycobacterium chelonae* infection treated with combined medication and surgical therapy: A case report

**DOI:** 10.1097/MD.0000000000046692

**Published:** 2025-12-26

**Authors:** Shuiling Li, Minghai Zhang

**Affiliations:** a Chaohu Hospital Affiliated to Anhui Medical University, Hefei, Anhui Province, China.

**Keywords:** diagnosis, MC, medication therapy, surgical treatment

## Abstract

**Rationale:**

The misuse of antibiotics and immunosuppressive agents has led to an increase in the incidence of infections caused by opportunistic pathogens, and even rare microbial infections such as *Mycobacterium chelonae* (MC) have emerged. Currently, there are very few reported cases of MC infection globally, and clinical experience in treating this disease is limited. This case report focuses on the treatment methods for infections caused by this bacterium, aiming to accumulate experience for clinicians in the diagnosis and treatment of this condition.

**Patient concerns:**

This study reports a case of MC infection that occurred following an invasive cosmetic injection treatment. Due to the progression of the condition, medical intervention was required. The patient underwent combined medication and surgical therapy, and the treatment outcomes were subsequently tracked.

**Diagnoses:**

Histopathological examination of the skin lesion, fungal culture, and metagenomic detection of the infectious pathogen suggested MC infection.

**Interventions:**

The patient underwent excision of the skin lesion, along with intravenous infusion of levofloxacin hydrochloride and sodium chloride injection at a dose of 0.4 g once daily and oral administration of rifampicin capsules at a dose of 0.45 g once daily. Complete lesion resolution was achieved at 1 month postoperatively.

**Outcomes:**

At the 1-month postoperative follow-up, the patient’s rash had healed completely.

**Lessons:**

This case fills a gap in domestic and international reports of MC infection following cosmetic filler injections. By employing metagenomic next-generation sequencing, the pathogen was rapidly identified within a short timeframe, significantly reducing the diagnostic delay associated with conventional bacterial culture and identification methods. Early radical lesion excision combined with a dual-antibiotic regimen (levofloxacin–rifampin) achieved complete resolution within 1 month, establishing a replicable treatment paradigm for cutaneous MC infections. Further multicenter prospective studies are warranted to optimize surgical *margins* and antimicrobial treatment duration.

## 1. Introduction

Recently, the widespread use of broad-spectrum antibiotics and immunosuppressive agents, the frequent implementation of invasive diagnostic and therapeutic procedures, and the increasing number of individuals with immunodeficiencies have led to a significant increase in infections caused by opportunistic pathogens. Among these pathogens, Mycobacterium chelonae (MC) is an opportunistic pathogen that is closely associated with the use of immunosuppressive agents, immunocompromised states, and invasive procedures such as surgery and injections.^[1,2]^ With the rapid rise of the beauty industry, the number of infection cases related to it has also increased year by year. There have been reports of infections caused by procedures such as cosmetic injections and liposuction.^[3]^ MC infection often affects single or multiple sites, including the lungs, skin, and subcutaneous tissues.^[4]^ The clinical manifestations of MC infection are usually nonspecific and can easily be misdiagnosed as other skin or soft tissue infections, leading to delayed treatment. Although the overall isolation rate of rapidly growing nontuberculous mycobacteria is increasing, cutaneous and soft tissue infections caused by MC remain rare, with only a limited number of confirmed cases reported worldwide. Current guidelines merely provide generalized recommendations such as “abscess excision combined with macrolides or multidrug therapy,”^[5]^ while evidence-based data on surgical timing, resection margins, and optimal postoperative antibiotic combinations and duration remain virtually nonexistent. This case report is the first to focus on a combined medical-surgical approach for localized cutaneous MC infection. Rapid preoperative diagnosis was achieved via metagenomic next-generation sequencing (mNGS), and favorable outcomes were obtained using an ``early radical excision + levofloxacin–rifampin combination’’ regimen. Our findings aim to provide new practical evidence for the combined pharmaco-surgical management of MC skin infections.

## 2. Case summary

*Patient information*: gender: female. Age: 44 years old. Residence: Ma’anshan, Anhui Province Chief Complaint: multiple cysts on both temporal and frontal regions for 2 months. History of present illness: 2 months ago, the patient developed multiple cysts the size of soybeans on both temporal and frontal regions without any obvious cause. The cysts were painless and non-pruritic, with a light red appearance, firm texture, and clear margins. The patient did not pay much attention initially. However, she later noticed that the cysts gradually enlarged, softened, and coalesced, accompanied by pain and mild itching. She then visited a local health clinic, where she was treated with intravenous anti-inflammatory fluids (details unknown) and oral cephalosporin antibiotics. After 10 days of treatment, the cysts showed some improvement, and she stopped the medication on her own. Subsequently, the cysts reappeared. Two days ago, a cyst on the right temporal region ruptured, discharging purulent and bloody secretions, followed by a reduction in size and the appearance of white scales on the surface. The patient’s symptoms have not improved, prompting her to visit our hospital’s outpatient department. She was admitted with a provisional diagnosis of “cystic acne.” Further history: before the onset of the rash, the patient had received wrinkle-reducing injections in the temporal and frontal regions (specific drugs unknown). Since the onset of the disease, the patient has had normal mental status, appetite, and sleep. Her bowel movements and urination have been regular, and there has been no significant change in body weight. The patient has been previously healthy, with no history of food or drug allergies, hypertension, or diabetes.

*Physical examination vital signs*: temperature: 36.4 °C, pulse: 70 beats per minute, respiration: 17 breaths per minute, blood pressure: 136/95 mm Hg, weight: 75 kg.

**Figure 1.**
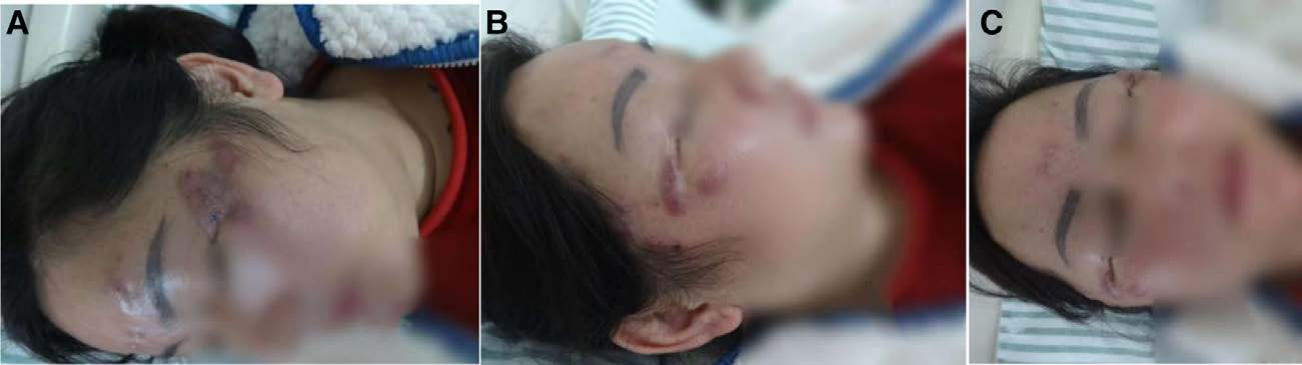
(A–C) Skin lesions on the forehead and temporal regions in a patient with MC infection.

*Specialist examination*: multiple cysts ranging in size from soybeans to pigeon eggs were observed on both temporal regions, with varying textures from soft to firm, and a dark red appearance. Some cysts were coalescent. The cyst on the left side measured approximately 3.5 × 2 cm and had a softer texture. The cyst on the right side had ruptured, was level with the skin, and had a size of about 2.5 × 2 cm, with a few white scales on the surface. Scattered cysts ranging in size from soybeans to broad beans were also present on the forehead. No tenderness was noted on palpation (see Fig. 1).

**Figure 2.**
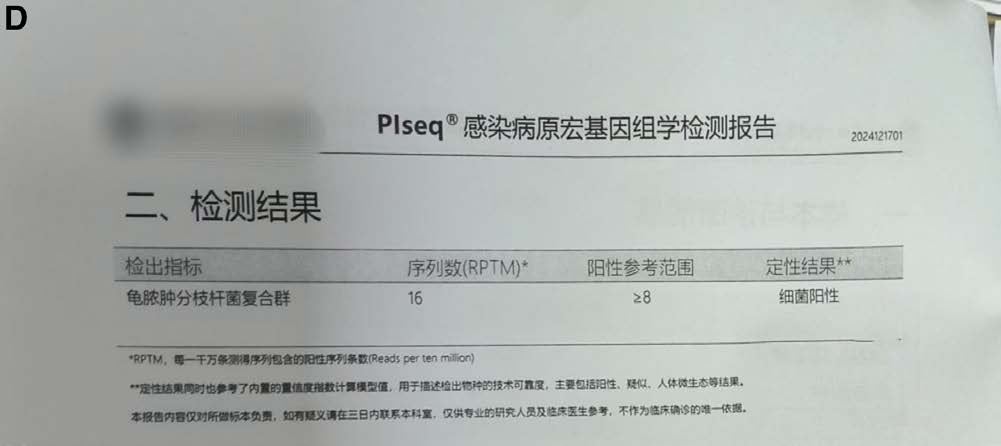
(D) Metagenomic sequencing results of the infectious pathogens in the frontal-temporal skin lesion tissue.

**Figure 3.**
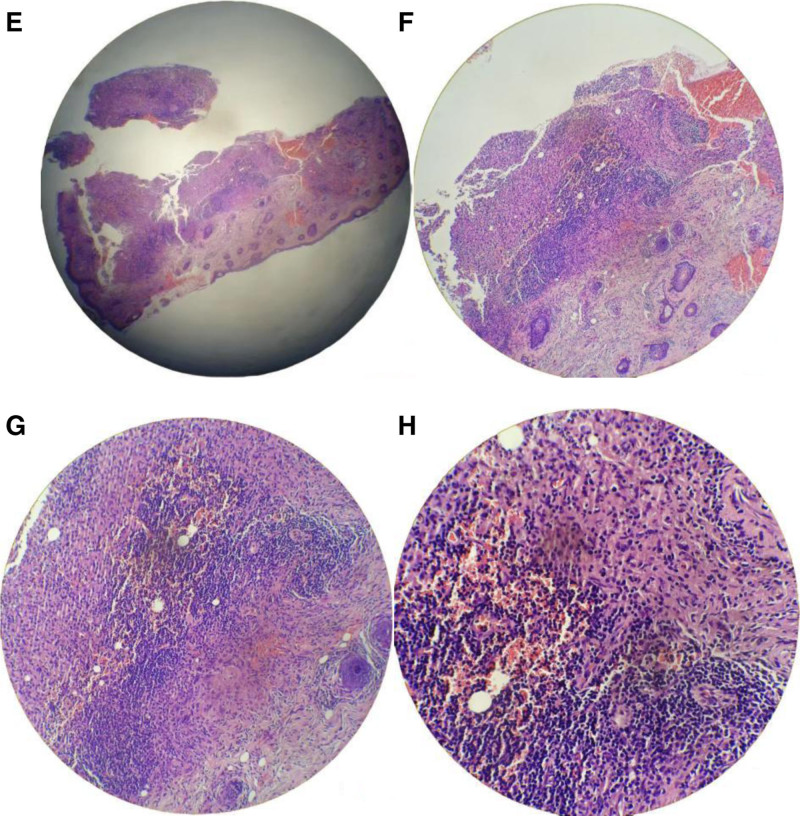
(E–H) Histopathology of the skin lesion shows chronic suppurative inflammation (HE, E: ×100; F: ×400).

*Laboratory and ancillary examinations*: routine blood tests, electrolytes, comprehensive biochemical profile, coagulation function, urinalysis, stool examination, preoperative panel, interleukin levels, acid-fast bacillus smear test, sex hormone panel (3 items), antinuclear antibody test, chest X-ray, and electrocardiogram showed no significant abnormalities. The erythrocyte sedimentation rate was 28 mm/h (reference range 0–21). mNGS was positive, with 16 sequences detected for the *Mycobacterium chelonae*–abscessus complex (see Fig. 2). Histopathological examination of the skin lesions revealed chronic suppurative inflammation of the subcutaneous soft tissue in the bilateral temporal and frontal regions (see Fig. 3).

*Diagnosis*: infection with MC in the frontal-temporal region.

*Therapeutic regimen*: intravenous infusion of levofloxacin hydrochloride and sodium chloride injection at a dose of 0.4 g once daily, in combination with oral administration of rifampicin capsules at a dose of 0.45 g once daily. Based on microbiological findings, the antimicrobial regimen was switched to clarithromycin 0.5 g every 12 hours plus doxycycline 0.1 g twice daily.

*Physical therapy*: red light irradiation of the skin lesion once daily.

*Surgical treatment*: excision of skin lesions. For smaller lesions, direct closure was performed after excision, while for larger lesions, an incision method was used. After excising the internal scar tissue and debriding the secretions, a mixture of isoniazid and amikacin was injected locally, followed by placement of petrolatum gauze for drainage.

*Prognosis*: 1 month after treatment, the patient was followed up and the rash had significantly improved.

The detailed diagnostic and therapeutic course is presented in Table 1.

**Table 1:** Patient’s clinical course and management timeline.

Time	Clinical manifestations	Primary intervention	Primary outcome
Before October 2024	Botulinum toxin injection to the temporal and frontal regions for wrinkle reduction	–	–
October 2024	Multiple cysts developed bilaterally in the temporal and frontal regions.	Intravenous anti-inflammatory infusion (agent unspecified) plus oral cephalosporin therapy	Transient regression of the cysts was followed by prompt recurrence.
December 7, 2024	Ulceration occurred over the right temporal cyst.	–	Purulent, blood-tinged discharge was observed.
December 9, 2024	Admission was planned under the working diagnosis of “cystic acne”; relevant investigations were to be completed.	Empirical antimicrobial therapy was initiated with intravenous levofloxacin 0.4 g once daily, oral rifampicin 0.45 g once daily, and daily red-light irradiation of the lesional skin.	Inflammation was brought under control.
December 11, 2024	Acid-fast bacilli smear microscopy for “Mycobacterium tuberculosis”	Negative	–
December 13, 2024	Excision of the cutaneous lesion was performed under local anesthesia.	Under local infiltration anesthesia, 10 facial lesions were excised. Lesions < 2.0 cm were removed and primarily closed; lesions ≥ 2.0 cm were incised and drained. The excision margin was set 0.3 cm beyond the visible lesion, extending to the subcutaneous fat. Internal scar tissue was excised and purulent material evacuated. The wound bed was then infiltrated with a mixture of isoniazid and amikacin, and a petroleum-impregnated gauze drain was placed.	Postoperative compression dressing was applied, and vital signs remained stable.
December 14, 2024	Postoperative wound dressing changes	A solution containing isoniazid and amikacin was infiltrated locally into the wound bed.	The surgical incision showed mild erythema and edema; scant purulent fluid was noted on the drain, with no evidence of active bleeding.
December 17, 2024	Histopathological examination of the lesional tissue	Chronic suppurative inflammation of the subcutaneous soft tissue in the bilateral temporal and frontal regions	Antimicrobial therapy was continued.
December 18, 2024	mNGS identified 16 sequence reads mapping to the MC complex.	Based on microbiological findings, the antimicrobial regimen was switched to clarithromycin 0.5 g every 12 hours plus doxycycline 0.1 g twice daily.	Pathogen was microbiologically confirmed.
After hospital discharge	Regular outpatient follow-up.	Outpatient wound-dressing changes, suture removal, and continuation of antimicrobial therapy.	Complete lesion resolution was achieved at 1 month postoperatively.

mNGS = metagenomic next-generation sequencing, MC = *Mycobacterium chelonae*.

Diagnostic basis and differential diagnosis:

1. Key evidence supporting MC infection:
2. * Epidemiological history*: the patient had a documented history of cosmetic injections (temporal/frontal wrinkle-reducing injections) prior to symptom onset, and such aesthetic medical procedures are a well-established risk factor for cutaneous nontuberculous mycobacterial (NTM) infections.
3. * Clinical characteristics*: key differential diagnosis from cystic acne: while acne vulgaris typically presents with comedones/blackheads and predominantly affects sebaceous gland-rich areas (e.g., midfacial region), the current case demonstrated lesions strictly localized to injection sites (temporal/frontal regions) without comedone formation.

*Key differential diagnosis from bacterial abscess*: unlike typical bacterial infections (e.g., *Staphylococcus aureus*) which demonstrate rapid progression with purulent yellow exudate and good response to antibiotics (e.g., cephalosporins), the present case showed poor cephalosporin response and sanguinopurulent discharge characteristics.

(iii) *Laboratory confirmation: mNGS*: detected specific sequences of the *Mycobacterium abscessus* complex (16 reads), demonstrating higher sensitivity and specificity for NTM compared to conventional culture methods.

*Histopathology*: revealed chronic suppurative inflammation with granuloma formation, consistent with NTM infection. This pattern markedly differed from the follicular keratin plugs and sebaceous gland dilation characteristic of cystic acne.

1. Evidence ruling out alternative infections:
2. * Fungal infection*: the patient had no history of immunosuppression. Histopathology revealed no hyphae or spores, and mNGS detected no fungal sequences.
3. * Tuberculosis*: acid-fast bacillus smear was negative, histopathology showed no caseous necrosis, and chest X-ray findings were unremarkable.
4. *Inflammatory markers*: the mildly elevated erythrocyte sedimentation rate (28 mm/h) was consistent with the chronic inflammatory process characteristic of NTM infection.

## 3. Discussion

NTM are defined as mycobacteria other than *Mycobacterium tuberculosis* and *Mycobacterium leprae*, and include species such as MC, *Mycobacterium fortuitum*, and *Mycobacterium abscessus*.^[6]^ MC is widely distributed in natural environments such as water and soil, and possesses strong survival capabilities and adaptability. It is classified as a rapidly growing NTM in the Runyon classification system, belonging to Group IV.^[4,5]^ Cases of MC infection were first reported abroad in the 1970s,^[4]^ and subsequently, similar clinical infection cases have been reported domestically.^[7]^ Skin damage is the primary cause of MC skin infection, which can result from surgical procedures, injections, trauma, tattoos, and acupuncture, among others.^[8]^ Infections caused by this bacterium often manifest as erythema, nodules, ulcers with pain, and are prone to misdiagnosis as other cutaneous and soft tissue infections, leading to delayed treatment. The histopathological manifestations of MC skin infections are nonspecific. In the early stages, acute inflammation mediated by neutrophils is primarily observed. Subsequently, ill-defined granulomas may form, and necrotic foci may be present in some cases. In the late stages of the lesion, chronic suppurative inflammation is commonly seen, with the formation of small abscesses.^[9]^ The current diagnostic methods for MC infection mainly include direct smear microscopy, bacterial culture, biochemical reactions, antigen–antibody detection, molecular biology techniques, and metagenomic sequencing.^[10–12]^

In the treatment of MC infection, it is recommended to perform antimicrobial susceptibility testing first to clarify the drug sensitivity of the pathogen. Based on the results of the susceptibility test, sensitive antibiotics should be selected for targeted therapy, thereby improving the therapeutic effect and reducing the risk of drug resistance. According to the 2024 Expert Consensus on the Diagnosis and Treatment of Cutaneous NTM in China, MC is susceptible to linezolid, clarithromycin, and tobramycin, and it shows intermediate susceptibility to amikacin and ciprofloxacin.^[13]^ In the selection of drugs and the arrangement of treatment courses for MC infection, it is recommended to use at least 2 sensitive drugs in combination. The treatment course usually lasts for no <4 months. The specific duration should be adjusted according to the patient’s clinical response and the results of pathogen retesting.^[13]^

In contrast to the commonly reported treatment strategies in the international literature, the core management approach in this case was not solely reliant on pharmacological therapy, but rather adopted a combined surgical and medical regimen. For cutaneous soft tissue MC infection, German researchers Uslu et al.^[14]^ employed a triple-drug therapy consisting of surgical debridement in combination with clarithromycin, tobramycin, and imipenem. All 5 patients in their study achieved lesion healing within 2 years, with no recurrence observed during the 12-month follow-up period. In comparison, the majority of domestic and international reports describe the treatment of cutaneous MC infection primarily with antibiotics alone, which is associated with high drug exposure, prolonged treatment duration, and an increased risk of adverse effects.^[13,15]^ To the best of our knowledge, there have been no reports on a treatment approach relying exclusively on surgical intervention. This study rapidly identified the pathogen through mNGS technology, aligning with the recent trend towards molecular diagnostics,^[11]^ and significantly reduced the time to diagnosis compared to traditional culture methods. The antibiotic treatment strategy in this case was dynamically adjusted based on disease progression and diagnostic clarity: initially, levofloxacin was empirically selected for suspected bacterial infection (similar to the regimen reported by Ren Lulu et al^[11]^), leveraging its broad-spectrum antibacterial activity and anti-inflammatory effects to cover common skin pathogens. As the patient showed inadequate response to conventional antibiotics (e.g., cephalosporins) and suspicion of NTM infection related to cosmetic injections increased, local injections of isoniazid and amikacin were administered during surgical intervention to enhance drug concentration at the lesion site. Finally, based on MC abscessus complex detection via mNGS and recommendations from the Chinese Expert Consensus on Diagnosis and Treatment of Cutaneous Non-Tuberculous Mycobacterial Diseases (2024 Edition), the regimen was adjusted to clarithromycin combined with doxycycline. This decision was based on 3 considerations: first, mNGS technology provided rapid molecular etiological evidence for targeted therapy; second, the consensus indicates that MC is generally susceptible to macrolides and tetracyclines, and this combination regimen offers broad antimicrobial coverage, favorable safety profile, and good patient compliance; third, the significant clinical outcome (complete resolution of skin lesions after 1 month of treatment) confirmed its effectiveness, which is consistent with the efficacy of clarithromycin-based regimens reported by Peng Yiran et al^[16]^ and Yue Wuyang.^[17]^ Although the inability to provide conventional drug susceptibility testing results due to prolonged culture time and nonroutine clinical testing represents a limitation in this case, the treatment strategy strictly adhered to authoritative guidelines and achieved a successful outcome, offering a practical reference for empirical treatment based on molecular diagnostics and consensus guidelines in similar scenarios. Subsequent studies are recommended to incorporate drug susceptibility testing whenever possible to further optimize treatment strategies. Surgical intervention demonstrated unique clinical value in this case. The patient underwent excision of the facial skin lesion under local anesthesia. Given the facial location of the lesion, an excessively wide excision margin could lead to increased tension and significant scarring, adversely affecting both appearance and function. Intraoperatively, the lesion was resected 3 mm beyond the grossly visible border, reaching healthy dermal tissue. This margin width represented an individualized balance between radical removal and cosmetic preservation, rather than an arbitrary reduction. To our knowledge, no previous studies have reported surgical management of cutaneous MC infection or specified excision margins, nor is there a standardized approach. This case is the first to explicitly adopt a 3 mm margin and achieve immediate negative margins, thereby providing a feasible surgical paradigm for refractory cutaneous MC infections and offering preliminary evidence for future large-scale studies to determine optimal excision margins. Through abscess drainage combined with debridement, the procedure not only effectively removed necrotic tissue and significantly reduced bacterial load, but also yielded deep-tissue specimens, enabling subsequent pathogen confirmation via mNGS. Compared to pharmacotherapy alone, this combined strategy of surgery and targeted antibiotics addressed both acute infection control and aesthetic outcomes, resulting in rapid and complete clinical resolution. Owing to the patient’s refusal to continue follow-up after complete lesion healing, the observation period was limited to 1 month; consequently, late recurrence (which in Mycobacterium chelonae–abscessus group infections typically manifests 3–6 months after treatment cessation) could not be captured, and the long-term adverse effects associated with cumulative antibiotic exposure remain unevaluated.

In recent years, surgical debridement has been widely recognized as a key therapeutic approach to promote lesion healing in the treatment of rare microbial infections. Clinical studies have shown that for patients with infectious cysts, a comprehensive treatment plan combining surgical excision, postoperative drainage, and adjunctive antimicrobial therapy can effectively promote the repair and healing of damaged tissues. According to the research by Zhang Haigang et al.^[18]^ In the treatment of pediatric maxillary sinus infectious mucoceles, they innovatively adopted a surgical approach combining partial cystectomy with marsupialization. This method effectively improved the drainage function of the sinuses. Postoperatively, with standardized anti-infective treatment, the clinical symptoms of the patients were significantly alleviated. Follow-up examinations showed good prognosis, further validating the effectiveness of the combined surgical and pharmacological treatment strategy. Additionally, the 9th edition of Internal Medicine, published by People’s Medical Publishing House in Beijing, also points out that in the treatment of infective endocarditis, surgical debridement of infected tissues to restore valve function is a necessary therapeutic measure when the pathogen is a fungal infection, multidrug-resistant bacteria, or when complications such as abscesses or fistulas have formed. This indicates that in the treatment of complex infections, surgical intervention is not only an effective means of local lesion removal but also creates favorable conditions for subsequent pharmacological therapy, thereby significantly improving patient prognosis.

In the existing literature, there are relatively few reports of cases involving MC infection of the skin and subcutaneous tissue, especially those treated with a combination of medication and surgery. The patient in this case is an elderly woman who had received wrinkle-reducing injections (the specific drug is unknown) in the temporal and frontal regions prior to the onset of the rash. This may have provided favorable conditions for the growth and proliferation of MC. It is worth noting that the patient underwent surgical treatment, specifically excision of the skin lesions, in combination with medication therapy using levofloxacin and rifampicin. The treatment was highly effective. This suggests that a treatment regimen combining antimicrobial agents with surgical intervention may be one of the effective options for treating cysts caused by MC infection.

## 4. Describe

To protect patient privacy, all identifiable information was de-identified, and facial features in photographs were masked. Written informed consent was obtained from the patient (File S1, Supplemental Digital Content, https://links.lww.com/MD/Q934), authorizing the use of his/her clinical data under these privacy safeguards for the preparation and publication of this manuscript; the patient also reviewed and approved the final version of the manuscript. This retrospective case analysis utilized fully anonymized medical records; according to the Ethics Committee policy of Chaohu Hospital, Anhui Medical University, additional ethical approval was waived for anonymized retrospective studies.

## Author contributions

**Conceptualization:** Shuiling Li, Minghai Zhang.

**Data curation:** Shuiling Li, Minghai Zhang.

**Formal analysis:** Shuiling Li.

**Funding acquisition:** Shuiling Li.

**Investigation:** Shuiling Li.

**Methodology:** Shuiling Li.

**Project administration:** Shuiling Li.

**Resources:** Shuiling Li.

**Software:** Shuiling Li.

**Supervision:** Shuiling Li.

**Validation:** Shuiling Li.

**Visualization:** Shuiling Li.

**Writing – original draft:** Shuiling Li.

**Writing – review & editing:** Shuiling Li.

## Supplementary Material



## References

[1] do Carmo Barbosa BE Lacerda PN Campos LM. Nontuberculous mycobacteriosis (*Mycobacterium chelonae*): fatal outcome in a patient with severe systemic lupus erythematosus. An Bras Dermatol. 2023;98:878–81. 37407333 10.1016/j.abd.2022.12.005 PMC10589477

[2] Hammond SE Al-Bayati A Joumblat N Salgado CJ. *Mycobacterium chelonae* infection of the buttocks secondary to lipofilling: a case report and review of the literature. Aesthetic Plast Surg. 2017;41:1150–4. 28526906 10.1007/s00266-017-0890-3

[3] Rodriguez JM Xie YL Winthrop KL. *Mycobacterium chelonae* facial infections following injection of dermal filler. Aesthet Surg J. 2013;33:265–9. 23335647 10.1177/1090820X12471944 PMC4618382

[4] Gruft H Henning HG. Pulmonary mycobacteriosis due to rapidly growing acid-fast bacillus, *Mycobacterium chelonei*. Am Rev Respir Dis. 1972;105:618–20. 5017885 10.1164/arrd.1972.105.4.618

[5] Chinese Medical Association Tuberculosis Branch. Guidelines for the diagnosis and treatment of nontuberculous mycobacterial diseases (2020 Edition). Chin J Tuberc Respir Dis. 2020;43:918–46.

[6] Guglielmetti L Mougari F Lopes A Raskine L Cambau E. Human infections due to nontuberculous mycobacteria: the infectious diseases and clinical microbiology specialists’ point of view. Future Microbiol. 2015;10:1467–83. 26344005 10.2217/fmb.15.64

[7] Yongai L. Disseminated *Mycobacterium avium*-intracellular infection. International Medicine (Internal Medicine Section). 1984:52–3.

[8] Akram SM Rathish B Saleh D. *Mycobacterium chelonae* Infection [M/OL]//StatPearls. StatPearls Publishing; 2025. http://www.ncbi.nlm.nih.gov/books/NBK430806/. Accessed February 24, 2025. 28613557

[9] Gaudêncio M Carvalho A Bertão MI Barreiro I Bessa MI Gonçalves A. *Mycobacterium chelonae* cutaneous infection: a challenge for an internist. Eur J Case Rep Intern Med. 2021;8:003013. 34912746 10.12890/2021_003013 PMC8668006

[10] Zhongkui Z Daoxian K Taihua L. Molecular biological identification of *Mycobacterium avium* skin infection. Chin J Zoonoses. 2014;30:1125–8.

[11] Lulu R Hequn H Xing F. Diagnosis of *Mycobacterium chelonae* skin infection by metagenomic next-generation sequencing: a case report. Chin J Dermatol. 2024;38:1012–7.

[12] Qingtian L Yongzhang Z Yufeng Y. Report on the development of medical microbiology. Microbiol Bull. 2025;52:1–16.

[13] Mycobacteriosis Research Group of the China “Belt and Road” Dermatology Alliance, Division of Dermatology and STI Diagnosis, Chinese Association on Leprosy. Expert consensus on the diagnosis and treatment of cutaneous nontuberculous mycobacterial diseases in China (2024 Edition). Chin J Dermatol. 2024;57:109–18.

[14] Uslu U Böhm O Heppt F Sticherling M. Skin and soft tissue infections caused by *Mycobacterium chelonae*: more common than expected? Acta Derm Venereol. 2019;99:889–93. 31141157 10.2340/00015555-3230

[15] Meng FY Liang HY. Pharmacotherapy analysis of a postoperative intracerebral hemorrhage patient with suspected intracranial infection caused by *Mycobacterium chelonae*. Chin J Mod Appl Pharm. 2022;39:231–4.

[16] Yiran P Lele S Fangfang B. A case of *Mycobacterium chelonae* infection secondary to facial lupus erythematosus tumidus. China J Lepr Skin Dis. 2023;39:512–5.

[17] Wuyang Y Yan G Tongxin Li. Misdiagnosis of *Mycobacterium chelonae* infection as tuberculosis and leprosy: a case series analysis of two patients. Elec J Emerg Infect Dis. 2021;6:348–51.

[18] Haigang Z Hui’e Z Mingyue F. Endoscopic nasal surgery for maxillary sinus infected mucous cyst in children: a case report and literature review. J Clin Otolaryngol Head Neck Surg. 2025;39:268–71.

