# Supplementary material for: Mycobacterium chelonae infection treated with combined medication and surgical therapy: A case report
Source: Medicine (Baltimore). 2025 Dec 26;104(52):e46692. doi: 10.1097/MD.0000000000046692 (PMC12747043; doi:10.1097/MD.0000000000046692)
Supplement: Supplementary file 1 [file medi-104-e46692-s001.pdf]

Medicine®

PATIENT PHOTOGRAPHIC AUTHORIZATION, RELEASE AND DISCHARGE

I, Ma Yuqin, give my consent and authorize the photograph(s) and/or video featuring my likeness to be published in *Medicine®*, a Wolters Kluwer publication. I understand that such imaging records may be published by *Medicine®* and/or any party acting under the license and authority of *Medicine®* in any print, visual, electronic or broadcast media, specifically including, but not limited to, medical journals and textbooks, scientific presentations and teaching courses and Internet websites, for the purpose of informing the medical profession or the general public about plastic surgery methods, results, issues, trends, concerns and similar matters. I further understand that the imaging records shall become the property of *Medicine®*.

I understand that I may refuse to sign this authorization and such refusal will have no effect on the medical treatment I receive.

I release and discharge *Medicine®*, and all parties acting under their license and authority from all rights that I may have in the imaging records and from any claim that I may have relating to such use in publication, including any claim for payment in connection with distribution or publication of the imaging records in any medium or any claim arising from the distribution or publication by any third party.

I hereby warrant that I am over twenty-one years of age, and competent to contract in my own name.

I grant this consent as a voluntary contribution in the interest of public education and certify that I have read the above Authorization, Release and Discharge and fully understand its terms.

Patient Ma Yuqin Date 2018.03.24

WITNESS/PHYSICIAN: Lü Shuiling

I have read the above Authorization, Release, and Discharge. I am the parent, guardian or conservator of ______________, a minor. I am authorized to sign this consent on his/her behalf and I grant this consent as a voluntary contribution in the interest of public education.

Parent/Guardian ______________ Date ______________
